# Correlation between triglyceride-glucose index and early neurological deterioration in patients with acute mild ischemic stroke

**DOI:** 10.3389/fneur.2024.1441116

**Published:** 2024-11-28

**Authors:** Yang Liu, Zhiye Wang, Zuonian Zhang, Zhaomin Lu, Lihua Zhang, Wei Ding, Kai Fang, Xijin Pan, Mengyuan Ni, Junjun Liu

**Affiliations:** ^1^Department of Neurology, Nanjing Meishan Hospital, Nanjing, China; ^2^Department of Neurology, Drum Tower Hospital of Nanjing University, Nanjing, China; ^3^Department of Neuropsychiatry, Nanjing Meishan Hospital, Nanjing, China

**Keywords:** early neurological deterioration, acute ischemic stroke, mild, triglyceride glucose index, correlation

## Abstract

**Objective:**

The Triglyceride-glucose Index (TyG) index is a dependable metric for assessing the degree of insulin resistance, serving as a standalone predictor of ischemic stroke risk, but its precise relationship with early neurological deterioration (END) remains incompletely expounded within the context of acute mild ischemic stroke patients. This research is to examine the correlation of the TyG index with END among patients experiencing acute mild ischemic stroke in China.

**Methods:**

This retrospective analysis was conducted to systematically gather data regarding patients experiencing their maiden episode of acute mild ischemic stroke and hospitalized at the Neurology Department of Nanjing Meishan Hospital, located in Nanjing, Jiangsu Province, China, over the period extending from January 2020 to December 2022. The severity of stroke was determined through the utilization of the National Institutes of Health Stroke Scale (NIHSS) scores upon their admission. Demographic characteristics were collected, and measurements of fasting blood glucose, blood lipids, and glycosylated hemoglobin Alc levels were taken. END was defined as a one-point rise in the motor item function score on the NIHSS or a two-point increase in the overall score during the initial 72 h of hospitalization. For evaluating the correlation of the TyG index with END, a multivariate logistic regression analysis was carried out. To investigate whether there is a nonlinear relationship between the TyG index and END, smoothed curves were utilized.

**Results:**

The study included 402 patients diagnosed with acute mild ischemic stroke, with a mean age of 66.15 ± 10.04 years. Within this population, 205 were males (51.00%) and 197 were females (49.00%). Among these patients, 107 (26.62%) experienced END within 72 h of admission. Patients who developed END showed higher levels of the TyG index in comparison to those who remained stable (9.18 ± 0.46 vs. 8.87 ± 0.46, *p* < 0.001). In a comprehensive multivariate logistic regression analysis, the TyG index positively correlates with END (*OR* = 3.63, 95% CI: 1.75–7.54, *p* = 0.001). Furthermore, individuals in the fourth TyG index quartile exhibited a 2.36-fold heightened risk of END compared to those in the first quartile (95% CI: 1.38–8.19, *p* = 0.008). TyG index has a linear correlation with END in the generalized additive model (Log likelihood ratio test, *p* = 0.525).

**Conclusion:**

Our findings demonstrate that TyG index has a significant, independent, and positive correlation with END in Chinese individuals diagnosed with acute mild ischemic stroke. This underscores the TyG index’s potential usefulness as a valuable risk stratification tool for stroke patients.

## 1 Introduction

Ischemic stroke, the most prevalent subtype within the spectrum of stroke (1), ensues from perturbations in cerebral blood flow due to diverse etiologies. These disturbances ultimately result in an ischemic insult and subsequent hypoxic necrosis, which in turn lead to compromised cerebral function (2). Constituting a predominant proportion of ischemic stroke cases, mild ischemic strokes account for more than half of the afflicted patient population (3). Despite the initial presentation of minor symptomatic manifestations, individuals face the looming possibility of significant disability progression, with early neurological deterioration (END) serving as a pivotal harbinger of adverse prognostic outcomes (4). END refers to the deterioration of neurological faculties within a temporal window spanning from hours to days post-ischemic stroke onset. Most research defines this period as either 24 h or 72 h, with an incidence rate varying from 8.1 to 32.00% (5–7). This condition frequently results in prolonged hospitalization, a heightened risk of disability, and possibly fatal consequences (8, 9). Although efforts have been directed towards identifying prognostic markers of END in the overall population with ischemic stroke patients, limited attention has been accorded to those with mild strokes. Thus, elucidating the determinants associated with END in patients manifesting acute mild stroke is of paramount importance.

Multiple investigations have substantiated that hypertension, hyperglycemia, stroke subtype, hypercholesterolemia, hypertriglyceridemia, and inflammatory pathways augment the susceptibility to developing early neurological deterioration (END) (10, 11). Furthermore, insulin resistance (IR) is closely associated with ischemic stroke, potentially exacerbating END progression through diverse metabolic and inflammatory cascades (12, 13). IR denotes a state wherein the target tissues manifest diminished sensitivity to insulin’s physiological actions, culminating in attenuated biological responsiveness compared to normoinsulinemic conditions (14). Serving as the cardinal etiological factor in type 2 diabetes mellitus pathogenesis, IR constitutes a prevalent pathophysiological nexus underlying an array of metabolic perturbations (15). Conventional techniques for IR assessment, such as the hyperinsulinemic euglycemic clamp method and the homeostasis model assessment of insulin resistance (HOMA-IR), although efficacious, are encumbered by complexity and prohibitive costs, thus limiting their utility in large-scale population screenings (16). In contrast, the triglyceride glucose (TyG) index, which combines fasting triglyceride (TG) and fasting plasma glucose (FPG) values, is praised for its simplicity, cost-efficiency, and precision in gauging IR status (17–19). An accumulating body of evidence indicates the predictive value of the TyG index for END (20, 21). However, this proposition remains subject to debate among scholars (22–24). For instance, Zhang et al. (20) demonstrated that an elevated TyG index is positively linked to a higher likelihood of END, coupled with a lower chance of early neurological recovery, among individuals experiencing acute ischemic stroke after treatment with intravenous thrombolysis. Nevertheless, an alternate investigation failed to observe a similar relationship in patients with mild stroke undergoing intravenous thrombolysis (22). Furthermore, the clinical outcomes of stroke patients did not seem to be significantly influenced by the TyG index, regardless of BMI, according to Hou et al. (23). Alternative research endeavors have indicated the absence of a definitive correlation, potentially stemming from methodological disparities, variations in participant demographics, and differences in sample sizes (24).

Earlier investigations revealed that a raised TyG index is positively associated with an enhanced likelihood of END occurrence among ischemic stroke patients, notwithstanding the presence of varying and, at times, conflicting evidence. Nonetheless, as far as we are aware, no previous exploration has carried out a thorough analysis of how the TyG index relates to END specifically in individuals enduring acute mild ischemic stroke, particularly within the Chinese demographic. Our current investigation is primarily centered on patients experiencing a first-ever episode of acute mild ischemic stroke without prior pharmacological interventions, and all patients were treated with both aspirin and clopidogrel tablets following this visit, thereby providing a unique platform to scrutinize the interplay between the TyG index and END without interference from lifestyle adjustments or pharmaceutical treatments. This methodological approach is tailored to mitigate potential confounding factors such as disease progression and comorbidities, thereby bolstering the robustness of our findings. Thus, our foremost research objective lies in elucidating any potential inherent relationship linking the TyG index to END among a specific group of Chinese individuals afflicted by acute mild ischemic stroke, ultimately striving to establish a more scientifically rigorous and precise framework for clinical diagnosis and therapeutic interventions within this specialized medical realm.

## 2 Materials and methods

### 2.1 Study design and participants

A retrospective analysis was undertaken to systematically collect data pertaining to individuals experiencing their initial episode of acute mild ischemic stroke and hospitalized at the Neurology Department of Nanjing Meishan Hospital, situated in Nanjing, Jiangsu Province, China, spanning from January 2020 to December 2022. Strict standards for both including and excluding were imposed to ensure the integrity of the investigation. Specifically, eligible participants were mandated to be 18 years of age or older, possess a confirmed diagnosis of acute ischemic stroke devoid of prior pharmacological interventions (antiplatelet aggregation and/or anticoagulation and/or lipid-lowering therapy), exhibit no antecedent history of ischemic stroke, experience symptom onset within 72 h of presentation. Drawing on the CHANCE (25) and POINT (26) studies, this research defined mild stroke as having an NHISS score no higher than 3 upon admission. The findings of these studies indicate that early combination of aspirin and clopidogrel therapy significantly decrease the likelihood of recurrent ischemic stroke. Therefore, to ensure consistency and minimize the influence of medication on results, all patients participating in this study were administered a double antiplatelet therapy consisting of aspirin and clopidogrel. Patients who underwent thrombolysis or mechanical thrombectomy, as well as those afflicted by severe infectious ailments, hepatic or renal dysfunction, malignancies, hematological disorders, or incomplete medical documentation, were not included in the study. This study was conducted retrospectively, and all data were anonymized, thereby rendering informed consent unnecessary. The Ethics Committee of Nanjing Meishan Hospital granted authorization for the conduct of the research, ensuring ethical compliance (Approval No.: MSYYLL202312).

### 2.2 Baseline data collection

Upon admission, a comprehensive array of clinical parameters was compiled, encompassing demographic factors like age and gender, alongside physiological metrics such as body mass index (BMI), past occurrences of hypertension and diabetes mellitus, smoking patterns, alcohol intake, systolic blood pressure (SBP), and diastolic blood pressure (DBP). A tape measure was utilized for the purpose of conducting height assessments, affording a precision of 0.1 centimeters, while weight measurements were obtained using a calibrated platform scale accurate to 0.1 kg. BMI is calculated by taking the individual’s weight in kilograms and dividing it by the square of their height in meters. Blood pressure (BP) readings were obtained at least twice from the right upper arm utilizing a calibrated mercury sphygmomanometer with the participant in a seated position. When the difference between the two BP readings surpassed 5 mmHg, an additional measurement was conducted, and the mean of all three readings was documented. An individual classified as a ‘current smoker’ was one who, on an average basis, consumed at least one cigarette per day for over a year and continued to smoke within the past year (21). Similarly, a ‘current drinker’ was delineated as someone who had consumed an average of 100 milliliters of alcohol daily for more than a year and persisted in alcohol consumption nearly 1 year later (21).

Blood specimens were procured from the patient’s cubital vein subsequent to an 8-h fast. These samples underwent analysis to quantify levels of FBG, total cholesterol (TC), TG, high-density lipoprotein cholesterol (HDL-C), and low-density lipoprotein cholesterol (LDL-C) using the Beckman AU5821 Fully Automatic Chemistry Instrument. Additionally, glycosylated hemoglobin A1c (HbA1c) levels were determined utilizing the Wanfu Biodry Fluorescent Immunoanalyzer.

The severity of patients’ neurological status was evaluated using the NIHSS scoring system (3). To ensure accuracy and consistency in the NIHSS scoring procedure and minimize variability among evaluators, a standardized NIHSS assessment is performed solely by the same seasoned neurologist upon admission, as well as at the 24th and 72th hours subsequent to admission. The NIHSS encompasses 11 domains, namely consciousness level, gaze function, visual fields, facial paralysis, upper limb motility, lower limb motility, ataxia, sensation, speech, dysarthria, and neglect. It has a maximum score of 42, with higher scores correlating to more pronounced neurological impairment.

### 2.3 Definitions

The formula to determine the TyG index is: ln [fast TG (mg/dl) × FBG (mg/dl) / 2] (27). The quartile distribution of the TyG index values served as the basis for stratifying the patients into four distinct groups. END was delineated as an escalation of one point in the motor item function score of the NIHSS or a two-point elevation in the overall score (28), events that transpired within the initial 72 h subsequent to hospital admission.

### 2.4 Statistical analysis

The presentation of continuous variables was stratified based on their distribution characteristics. Specifically, variables adhering to a normal distribution were expressed using mean and standard deviation, with intergroup comparisons conducted via one-way ANOVA. The non-normal distribution of the data was visualized through the median and interquartile range, and the Kruskal-Wallis H test facilitated the analysis of intergroup comparisons. Categorical variables were expressed in terms of frequencies and percentages, and the Chi-square test was employed to evaluate the intergroup variations. An estimation of the relationship linking the TyG index to END was conducted via logistic regression models, considering both continuous and categorical representations derived from TyG index quartiles. Moreover, to determine the *p*-value for trend, the TyG index underwent a conversion, resulting in a categorical variable. Additionally, the independent variables’ multicollinearity was gauged by employing the Variance Inflation Factor (VIF), and any variables exceeding a VIF threshold of 5.0 were omitted from the final model. Each covariate was individually incorporated into the model, with covariates demonstrating a statistically significant association with END (*p* < 0.05) considered as potential confounders for inclusion in the final model. Three distinct models were constructed to ensure result consistency: Model I without adjustments, Model II adjusting for age and sex, and Model III adjusting for age, sex, diabetes mellitus, onset to initial assessment time, SBP, DBP, HbA1c, TC, and LDL-C. Aiming to delve into the non-linear relationship linking the TyG index to END, smoothing plots were employed for exploration. Receiver operating characteristic (ROC) curve analysis and calculation of the area under the curve (AUC) were used to evaluate the predictive ability of the TyG index for END.

The entire statistical analysis process was performed using the software suites known as R (available at http://www.r-project.org, courtesy of The R Foundation) and EmpowerStats (offered at http://www.empowerstats.com, provided by X&Y Solution, Inc., headquartered in Boston, Massachusetts, United States). In order to regulate the significance level in pairwise comparisons, the Bonferroni method was employed. Statistical significance was established by applying a two-tailed *p*-value cut-off of <0.05.

## 3 Results

### 3.1 Baseline characteristics

This investigation enrolled a group comprising 402 individuals diagnosed with acute mild ischemic stroke, characterized by a mean age of 66.15 ± 10.04. Among them, 205 were male (51.00%) and 197 were female (49.00%). Within this investigation, 107 patients (26.62%) exhibited END within 72 h of hospitalization. A notable finding revealed that patients experiencing END demonstrated elevated TyG index levels compared to their non-END counterparts (9.18 ± 0.46 vs. 8.87 ± 0.46, *p* < 0.001). Based on quartiles of TyG index, the individuals were stratified into four distinct groups as follows: The Q1 group (*n* = 103) comprised individuals with a TyG index below 8.60 (mean value: 8.35 ± 0.20), the Q2 group (*n* = 101) encompassed those with a TyG index ranging from 8.60 to 8.96 (mean value: 8.79 ± 0.11), the Q3 group (*n* = 101) consisted of participants with a TyG index between 8.96 and 9.32 (mean value: 9.14 ± 0.11), and finally, the Q4 group (*n* = 96) encompassed those with a TyG index equal to or greater than 9.32 (mean value: 9.59 ± 0.20).

Remarkably, the incidence of END cases within these quartiles displayed a gradient, with 15 (14.00%), 20 (18.70%), 28 (26.20%), and 44 (41.10%) cases in Q1, Q2, Q3, and Q4 groups, respectively, showcasing statistically significant distinctions (*p* < 0.001). Furthermore, notable disparities between groups (*p* < 0.05) were detected with regard to BMI, SBP, DBP, FBG, HbA1c, TG, TC, LDL-C, HDL-C, and the prevalence of diabetes. These outcomes are elaborated on in Table 1.

**Table 1:** Baseline characteristics of participants.

Variables	Total (*n* = 401)	TyG index quartile				*P-*value
		Q1 (*n* = 103)	Q2 (*n* = 101)	Q3 (*n* = 101)	Q4 (*n* = 96)	
Age (years)	66.15 ± 10.04	65.43 ± 9.87	67.15 ± 10.86	66.28 ± 9.52	65.72 ± 9.92	0.631
Gender						0.867
Male	205 (51.00%)	49 (47.60%)	54 (52.90%)	53 (52.50%)	49 (51.00%)	
Female	197 (49.00%)	54 (52.40%)	47 (47.10%)	48 (47.50%)	47 (49.00%)	
BMI, kg/m^2^	24.05 ± 2.37	23.20 ± 2.16	23.86 ± 2.42	24.80 ± 2.35	24.37 ± 2.26	<0.001
Hypertension, *n* (%)	181 (45.00%)	40 (38.83%)	41 (40.59%)	49 (48.51%)	51 (53.13%)	0.133
Diabetes mellitus, *n* (%)	148 (36.80%)	6 (4.10%)	10 (6.80%)	54 (36.50%)	78 (52.70%)	<0.001
Current smoking, *n* (%)	147 (36.60%)	35 (23.80%)	35 (23.80%)	37 (25.20%)	40 (27.20%)	0.660
Current drinking, *n* (%)	99 (24.60%)	24 (24.20%)	27 (27.30%)	21 (21.20%)	27 (27.30%)	0.633
Onset to initial assessment time (hours)	20.00 (12.00, 26.00)	20.00 (12.00, 26.25)	20.00 (12.00, 26.50)	21.00 (13.00, 27.00)	20.00 (12.00, 26.00)	0.773
SBP, mmHg	150.13 ± 19.71	145.89 ± 18.84	147.75 ± 17.72	150.69 ± 19.86	156.61 ± 21.01	0.001
DBP, mmHg	82.42 ± 13.27	80.70 ± 11.37	81.63 ± 13.50	81.81 ± 14.30	85.75 ± 13.41	0.039
FBG, mmol	5.76 (4.98, 7.85)	4.81 (4.08, 5.35)	5.36 (4.85, 5.77)	6.49 (5.70, 7.85)	9.31 (7.87, 9.96)	<0.001
HbA1c, %	6.00 (5.80, 7.30)	5.70 (5.40, 6.00)	5.90 (5.75, 6.00)	6.60 (5.90, 7.40)	7.40 (6.80, 8.38)	<0.001
TG, mmol	1.62 (1.27, 1.89)	1.15 (1.07, 1.23)	1.56 (1.38, 1.75)	1.76 (1.55, 2.13)	2.00 (1.74, 2.44)	<0.001
TC, mmol	4.38 ± 0.79	4.11 ± 0.77	4.44 ± 0.75	4.39 ± 0.80	4.57 ± 0.77	<0.001
LDL-C, mmol	2.68 (2.30, 3.09)	2.54 (2.11, 3.07)	2.67 (2.32, 2.99)	2.65 (2.26, 3.25)	2.88 (2.44, 3.27)	0.014
HDL-C, mmol	1.11 (0.95, 1.31)	2.00 (1.00, 3.00)	2.00 (1.00, 3.00)	2.00 (1.00, 3.00)	2.00 (1.00, 3.00)	0.002
NIHSS, score	2.00 (1.00, 3.00)	2.00 (1.00, 3.00)	2.00 (1.00, 3.00)	2.00 (1.00, 3.00)	2.00 (1.00, 3.00)	0.698
END, *n* (%)	107 (26.60%)	15 (14.00%)	20 (18.70%)	28 (26.20%)	44 (41.10%)	<0.001

The variables are presented as *n* (%) or the mean ± SD or median (quartile 1, quartile 3), TyG: triglyceride glucose; BMI: body mass index; SBP: systolic blood pressure; DBP: diastolic blood pressure; FBG: fasting blood glucose; HbA1c: glycosylated hemoglobin Alc; TG: triglyceride; TC: total cholesterol; HDL-C, high-density lipoprotein cholesterol; LDL-C: low-density lipoprotein cholesterol; NIHSS: National Institutes of Health Stroke Scale; END: early neurological deterioration.

### 3.2 Associations between END and TyG

**Figure 1:**
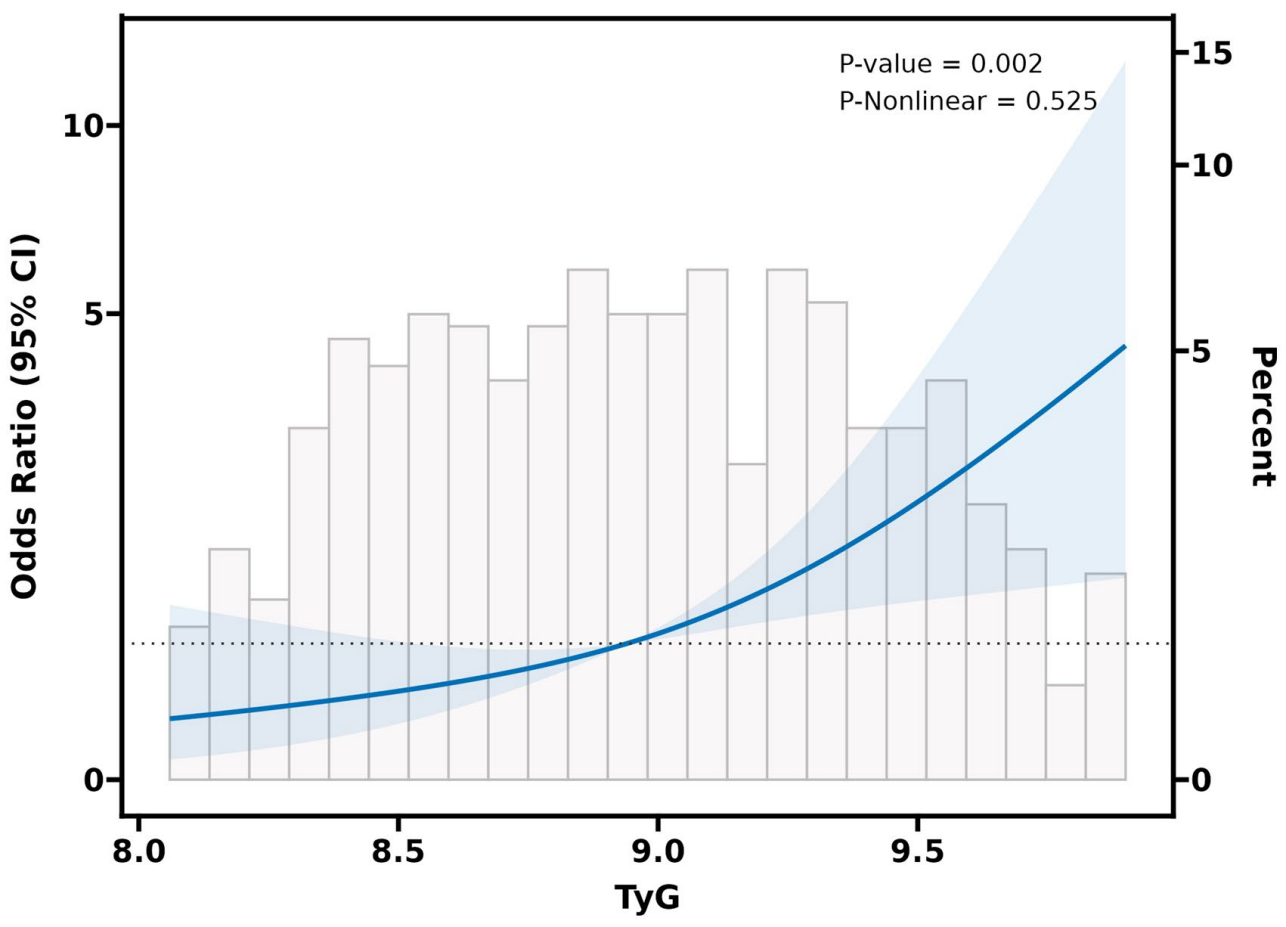
The relationship between TyG index and the probability of END. A linear relationship between TyG index and the probability of END was observed after adjusting for age, sex, diabetes mellitus, SBP, DBP, HbA1c, TC, LDL-C (Log likelihood ratio test, *p* = 0.525).

The outcomes of the univariate logistic regression analysis, as depicted in Table 2, underscored notable positive relationships (*p* < 0.05) between END and various factors, including age, diabetes, onset to initial assessment time, SBP, DBP, FBG, HbA1c, TG, TC, LDL-C, and the TyG index. Upon comprehensive adjustment for confounding variables, the multivariate logistic regression analysis, detailed in Table 3, uncovered a marked connection of higher TyG index levels with a heightened risk of END (*OR* = 3.63, 95% *CI*: 1.75 to 7.54, *p* = 0.001). Notably, patients belonging to the highest category of the TyG index exhibited a significantly increased tendency towards END in contrast to those in the lowest category, even after meticulous adjustment for multiple factors (*OR* = 3.36, 95% CI: 1.38 to 8.19, *p* = 0.008). Furthermore, employing generalized additive models, Figure 1 visually demonstrates a direct linear relationship linking the TyG index to END, rather than exhibiting a nonlinear relationship (Log likelihood ratio test, *p* = 0.525).

**Table 2:** Relationship of each variable and END in univariate analysis.

Variables	*β*	SE	Waldyχ^2^	*P*-value	OR (95%CI)
Age	0.025	0.011	4.584	0.032	1.03 (1.00 ~ 1.05)
Sex	−0.124	0.226	0.302	0.583	0.88 (0.57 ~ 1.38)
BMI	0.022	0.048	0.208	0.648	1.02 (0.93 ~ 1.12)
Hypertension,	0.298	0.226	1.739	0.187	1.35 (0.87 ~ 2.10)
Diabetes mellitus	0.779	0.23	11.441	0.001	2.18 (1.39 ~ 3.42)
Current smoking	0.156	0.232	0.453	0.501	1.17 (0.74 ~ 1.84)
Current drinking	0.112	0.259	0.187	0.666	1.12 (0.67 ~ 1.86)
Onset to initial assessment time	0.020	0.009	5.060	0.024	1.02 (1.00 ~ 1.04)
SBP	0.033	0.006	26.867	<0.001	1.03 (1.02 ~ 1.05)
BP	0.043	0.009	21.964	<0.001	1.04 (1.03 ~ 1.06)
FBG	0.261	0.052	25.523	<0.001	1.30 (1.17 ~ 1.44)
HbA1c	0.288	0.101	8.142	0.004	1.33 (1.09 ~ 1.63)
TG	0.919	0.249	13.598	<0.001	2.51 (1.54 ~ 4.08)
TC	0.541	0.15	13.077	<0.001	1.72 (1.28 ~ 2.30)
LDL-C	0.591	0.169	12.268	<0.001	1.81 (1.30 ~ 2.51)
HDL-C	−0.356	0.333	1.14	0.286	0.70 (0.37 ~ 1.35)
TyG	1.423	0.262	29.406	<0.001	4.15 (2.48 ~ 6.94)

TyG: triglyceride glucose; BMI: body mass index; SBP: systolic blood pressure; DBP: diastolic blood pressure; FBG: fasting blood glucose; HbA1c: glycosylated hemoglobin Alc; TG: triglyceride; TC: total cholesterol; HDL-C, high-density lipoprotein cholesterol; LDL-C: low-density lipoprotein cholesterol.

**Table 3:** Relationship between TyG index and END in different models.

变量	Model 1		Model 2		Model 3	
	OR(95% CI)	*P*-value	OR(95% CI)	*P*-value	OR(95% CI)	*P*-value
TyG index	4.15 (2.48 ~ 6.94)	<0.001	4.36 (2.57 ~ 7.38)	<0.001	3.63 (1.75 ~ 7.54)	0.001
TyG index quartile						
Q1 group	Reference	–	Reference	–	Reference	–
Q2 group	1.43 (0.69 ~ 2.98)	0.339	1.35 (0.65 ~ 2.83)	0.425	1.24 (0.57 ~ 2.67)	0.589
Q3 group	2.25 (1.12 ~ 4.53)	0.023	2.21 (1.09 ~ 4.47)	0.027	1.77 (0.80 ~ 3.91)	0.162
Q4 group	4.96 (2.52 ~ 9.79)	<0.001	5.02 (2.53 ~ 9.95)	<0.001	3.36 (1.38 ~ 8.19)	0.008
*p* for trend	<0.001		<0.001		0.008	

CI, confidence interval; Model I adjusted for none; Model II adjusted for age, sex; Model III adjusted for age, sex, diabetes mellitus, Onset to initial assessment time, SBP, DBP, HbA1c, TC, LDL-C.

### 3.3 Predictive ability of TyG index for END

**Figure 2:**
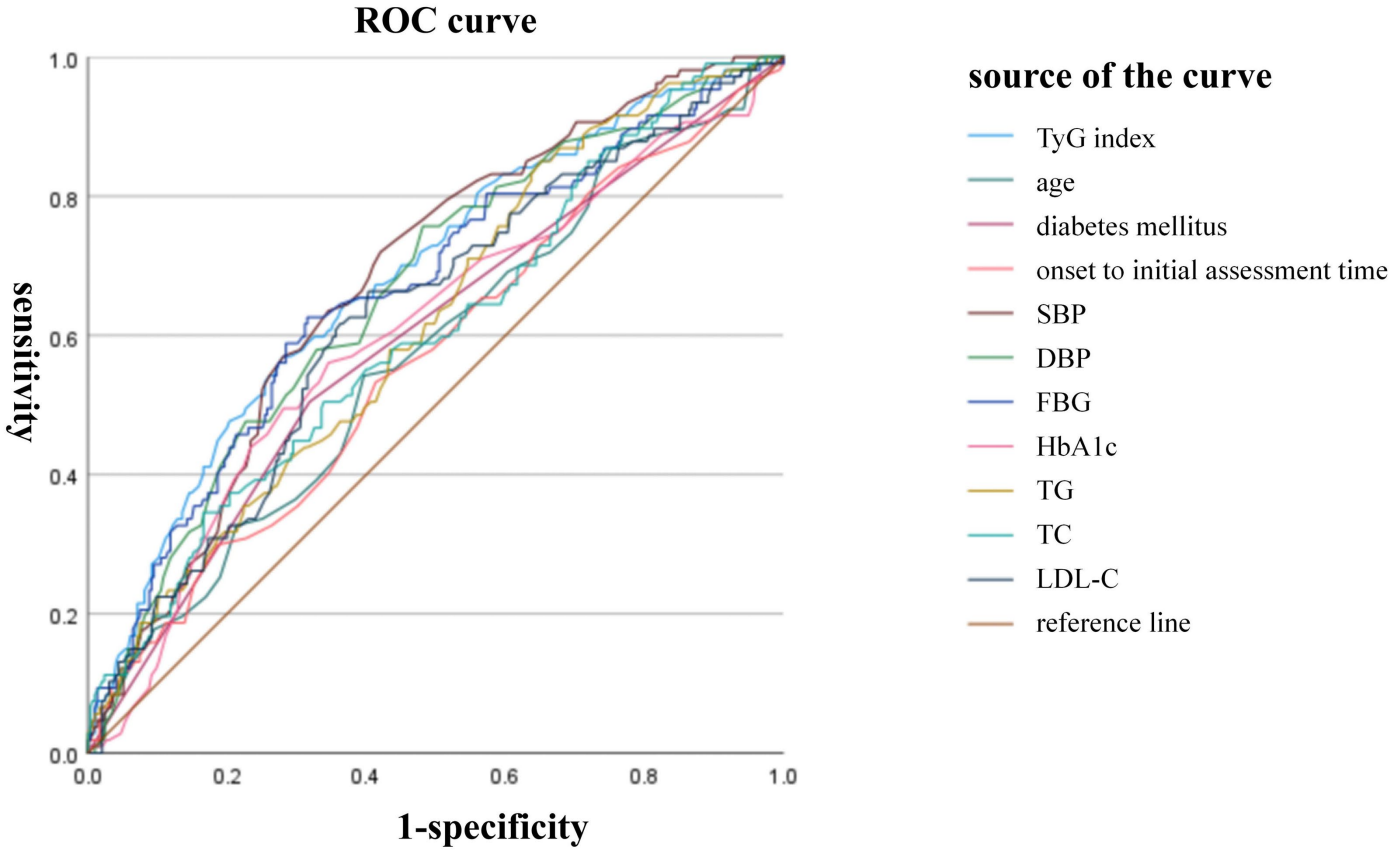
Predictive ability of TyG index for END. ROC curve analysis demonstrated that the TyG index has the ability to predict END (AUC = 0.683). The AUC value of other factors were: age (0.570), diabetes mellitus (0.593), onset to initial assessment time (0.564), SBP (0.679), DBP (0.664), FBG(0.662), HbA1c (0.602), TG(0.615), TC (0.604), and LDL-C (0.626).

As shown in Figure 2, ROC curve analysis demonstrated that the TyG index has the ability to predict END (AUC = 0.683), and the AUC value is higher than that of other factors with statistical significance in the univariate analysis (Table 2): age (0.570), diabetes mellitus (0.593), onset to initial assessment time (0.564), SBP (0.679), DBP (0.664), FBG(0.662), HbA1c (0.602), TG(0.615), TC (0.604), and LDL-C (0.626).

## 4 Discussion

To our best knowledge, this research marks the first foray into studying the correlation of the TyG index with END in Chinese individuals afflicted with acute mild ischemic stroke. Our research demonstrates that 26.62% of patients suffered from END after experiencing an acute mild ischemic stroke. Moreover, individuals who experienced END displayed higher TyG index levels, contrasting with those who did not. Remarkably, a higher prevalence of END was observed among patients falling within the highest category of the TyG index, in stark contrast to those belonging to the lowest category. Even upon adjustment for potential confounders, a significant positive linkage between the TyG index and END persisted. The consistency across all three logistic regression models underscores the robustness and reliability of our results. Furthermore, the smoothed curve illustrates a linear relationship between the TyG index and END. The revelations drawn from these findings offer profound understandings of the relationship between the TyG index and ischemic stroke. Nonetheless, further investigation is essential to elucidate the underlying mechanisms of this association, considering the array of potential pathways that may be involved.

Recent scientific inquiries have revealed a connection between the TyG index and the emergence as well as advancement of ischemic stroke (2, 29). A comprehensive meta-analysis revealed a significant positive correlation linking the TyG index to the occurrence of ischemic stroke (2). Individuals with elevated TyG index demonstrated a 150% heightened risk of recurrent stroke and a 1.4-fold greater probability of mortality (2). Furthermore, a longitudinal cohort study conducted by Huang et al. (29), encompassing 19,924 hypertensive individuals, revealed a strong correlation linking the TyG index directly to the occurrence of ischemic stroke, despite the absence of a meaningful link with hemorrhagic stroke.

Moreover, multiple investigations have unveiled a robust correlation linking the TyG index to the onset of END among individuals facing acute ischemic stroke. Throughout hospitalization, these individuals are confronted with the likelihood of END, which can culminate in unfavorable functional outcomes, prolonged hospital stays, cardiovascular complications, and heightened mortality hazards (30). A retrospective scrutiny involving 2,129 acute ischemic stroke patients, delineated as experiencing END if manifesting a two-point increment in the NIHSS score within 7 days post-admission, disclosed the TyG index as an autonomous predictor for END (31). Specifically, those belonging to the highest TyG index category exhibited a 5.906-fold augmented likelihood of END (95% CI: 3.676 to 9.488, *p* < 0.05) (31). Furthermore, a large-scale multicenter inquiry incorporating 3,216 acute ischemic stroke patients from 22 medical facilities, employing the modified Rankin scale (mRS) to gauge neurological function and defining unfavorable prognosis as an mRS score of 4–6 at discharge, established a linkage connecting the TyG index with both poor discharge prognoses and mortality during hospitalization (21). However, the correlation linking the TyG index to individuals experiencing mild ischemic stroke remains inadequately elucidated. The present investigation delineates that TyG index exhibits a profound linkage to END among individuals enduring acute mild ischemic stroke, thereby harmonizing with antecedent scholarly pursuits.

However, the clinical relevance of TyG in prognosticating ischemic stroke outcomes remains a contentious topic. A meta-analysis compiling information gathered across 18 separate investigations encompassing 592,635 ischemic stroke cases, unveiled a correlation between elevated TyG index and both stroke recurrence and mortality. Nevertheless, no significant link emerged between TyG index and adverse functional outcomes or neurological deterioration (24). Nam et al. (32) elucidated distinct associations between the TyG index and END across various subtypes of subcortical infarctions. Particularly in patients presenting with anterior subcortical infarctions, a noteworthy correlation remained evident concerning the TyG index and END, despite rigorous adjustments made to account for various influencing factors in the multivariate analysis (*OR* = 2.92, 95% *CI*: 1.35–6.29, *p* = 0.006) (32). Conversely, no such correlation was observed in patients presenting with distal subcortical ischemic stroke (32). Furthermore, the TyG index emerges as a standalone indicator of END onset in individuals with diabetes, whereas it lacks a similar impact in those without diabetes (32). The observed variation may stem from unexplained heterogeneity, influenced by several factors. These include the accessibility of effective secondary prevention, CYP2C19 genetic makeup, consistent rehabilitation practices, familial backing, the gravity of ischemic stroke, and the kind of acute phase therapy employed (25, 33–35). Furthermore, ethnic disparities, discrepancies in study design, sample size, and follow-up duration could also contribute to this heterogeneity. For instance, the definition of a mild stroke varied among studies, with most setting an NIHSS score threshold at either three (25, 26) or five (36, 37). Moreover, discrepancies existed in determining the timing of END. Some studies defined it within 24 h (38, 39), while others extended the window to 72 h post-stroke onset (40, 41). In this investigation, mild ischemic stroke was defined as an NIHSS score of three, with neurological deterioration within 72 h serving as the END criterion, yielding a rate of 26.62%. This finding concurs with rates reported by Liu et al. (42) and Boulenoir et al. (7). Given the ongoing debate surrounding the correlation linking the TyG index to ischemic stroke prognosis, further research is essential to delineate distinct risk stratification groups and establish a theoretical framework for targeted preventive measures. Future studies should employ robust cross-validation, larger cohorts, and collaborative multicenter approaches to deepen our understanding of this relationship.

Acute ischemic stroke may be triggered by IR reflected by the TyG index through various pathophysiological mechanisms, ultimately leading to the development of END. Primarily, IR has the potential to exacerbate endothelial dysfunction and stimulate platelet hyperactivation, thereby fostering the initiation and progression of atherosclerotic thrombosis (43). Secondly, IR can heighten the body’s oxidative stress response, resulting in the dysfunction of mitochondria (44). Thirdly, the elevation in the activity of matrix metalloproteinase 9 triggered by IR gives rise to a cascade of inflammatory reactions, ultimately leading to ischemia reperfusion injury effects in the brain (45). Additionally, IR can induce muscle sympathetic activity and catabolism, resulting in muscle atrophy and deterioration of motor function (46). Furthermore, the TyG index emerges as a superior indicator of cerebrovascular disease risk owing to its incorporation of both FBG and TG levels. Both hyperglycemia and hypertriglyceridemia have been implicated in increasing the likelihood of thrombotic events and exacerbating oxidative stress responses, potentially disrupting the integrity of the blood–brain barrier and exacerbating neurological manifestations (47, 48). Lastly, IR is a significant factor leading to elevated blood pressure (49), potentially causing more severe atherosclerosis and poorer collateral circulation. Although our study did not find differences in the prevalence of hypertension among different TyG index groups, the SBP and DBP were notably higher in the high TyG index group. The reasons for this phenomenon may include: high TyG levels leading to exacerbated systemic inflammation and impaired vascular endothelial function, which in turn cause an excessive increase in vascular reactivity (50); patients with high TyG index levels may require more aggressive antihypertensive treatment strategies and stricter lifestyle modifications to control their blood pressure; there may be differences in the distribution of hypertension subtypes across different TyG index groups (51) and a higher proportion of patients with END under stress in the high TyG index group. These factors acting together may result in significantly higher blood pressure levels in patients with high TyG index, even when the prevalence of hypertension is the same.

In this investigation, we applied multivariate logistic regression analysis and curve fitting techniques to initially establish a linear positive relationship linking the TyG index to the occurrence of END among individuals with acute mild ischemic stroke. This discovery furnishes valuable insights for forthcoming inquiries and deepens our comprehension of disease progression within this specific population of patients. Certain limitations warrant attention and refinement in subsequent research pursuits. Firstly, the dataset predominantly emanated from a solitary secondary hospital in China, potentially constraining the diversity of samples and the generalizability of findings across broader populations. Considering regional disparities, variations in hospital classifications, patient demographics, and other pertinent factors, the extrapolation of results to alternative medical settings or geographical regions may be compromised. To bolster the external validity of future investigations, concerted efforts should be directed towards procuring data from multiple medical establishments or diverse geographic locales, thereby ensuring a more inclusive and representative sample pool. Secondly, due to the retrospective character of this research, it was imperative to collect and organize past medical event-related data. This methodology is susceptible to selection bias, wherein researchers’ subjectivity in participant selection may result in an unrepresentative sample. Additionally, retrospective studies may encounter challenges related to recall bias and incomplete data documentation. For example, existing medical records have not included detailed information on the use of related medications of dyslipidemia, potentially compromising the accuracy of study outcomes. Therefore, forthcoming research endeavors should contemplate adopting a prospective cohort design to gather more thorough and precise data through prolonged patient monitoring and tracking, ultimately bolstering the internal validity of the research. Lastly, it is essential to acknowledge that the utilization of the TyG index in this investigation may be susceptible to potential interference from various variables, including recent consumption of glucose-lowering and lipid-regulating medications, as well as dietary patterns, among others. These factors have the potential to influence the measurement outcomes of the TyG index, thereby undermining the precision of the study’s findings. While efforts were made to minimize the impact of these confounding variables during data collection and analysis, their complete elimination remains challenging. Thus, future research should endeavor to delve deeper into unpacking the intricate connections linking the TyG index to diverse factors, thereby enhancing its precision in diagnosing and managing conditions such as diabetes.

In conclusion, a significant and substantive correlation has been uncovered connecting the TyG index to END among patients presenting who suffer from acute mild ischemic stroke. This correlation demonstrates a consistent linear trend of positive association. These findings present novel insights for clinical practitioners, underscoring the imperative to augment the evaluation of the TyG index in this specific population of patients. Such enhancements can enable clinicians to more precisely evaluate the patient’s status and formulate customized therapeutic strategies.

## Data Availability

The raw data supporting the conclusions of this article will be made available by the authors, without undue reservation.
